# Eye Movements in Ephedrone-Induced Parkinsonism

**DOI:** 10.1371/journal.pone.0104784

**Published:** 2014-08-12

**Authors:** Cecilia Bonnet, Jan Rusz, Marika Megrelishvili, Tomáš Sieger, Olga Matoušková, Michael Okujava, Hana Brožová, Tomáš Nikolai, Jaromír Hanuška, Mariam Kapianidze, Nina Mikeladze, Nazi Botchorishvili, Irine Khatiashvili, Marina Janelidze, Tereza Serranová, Ondřej Fiala, Jan Roth, Jonas Bergquist, Robert Jech, Sophie Rivaud-Péchoux, Bertrand Gaymard, Evžen Růžička

**Affiliations:** 1 Department of Neurology and Centre of Clinical Neuroscience, Charles University in Prague, 1st Faculty of Medicine and General University Hospital, Prague, Czech Republic; 2 Department of Circuit Theory, Faculty of Electrical Engineering, Czech Technical University, Prague, Czech Republic; 3 Department of Neurology, S. Khechinashvili University Clinic, Tbilisi, Georgia; 4 Institute of Medical Research, Ilia State University, Tbilisi, Georgia; 5 Department of Cybernetics, Faculty of Electrical Engineering, Czech Technical University, Prague, Czech Republic; 6 Research Institute of Clinical Medicine, Tbilisi, Georgia; 7 Institute of Pharmacology, First Faculty of Medicine, Charles University in Prague and General University Hospital in Prague, Prague, Czech Republic; 8 Analytical Chemistry and Neurochemistry, Department of Chemistry, Biomedical Center and SciLife Lab, Uppsala University, Uppsala, Sweden; 9 CRICM UPMC/INSERM UMR_S975, CNRS UMR7225, ICM, Pitié-Salpêtrière Hospital, Paris, France; 10 Pierre et Marie Curie Paris-6 University, Paris, France; Florey Institute of Neuroscience and Mental Health, The University of Melbourne, Australia

## Abstract

Patients with ephedrone parkinsonism (EP) show a complex, rapidly progressive, irreversible, and levodopa non-responsive parkinsonian and dystonic syndrome due to manganese intoxication. Eye movements may help to differentiate parkinsonian syndromes providing insights into which brain networks are affected in the underlying disease, but they have never been systematically studied in EP. Horizontal and vertical eye movements were recorded in 28 EP and compared to 21 Parkinson's disease (PD) patients, and 27 age- and gender-matched healthy subjects using standardized oculomotor tasks with infrared videooculography. EP patients showed slow and hypometric horizontal saccades, an increased occurrence of square wave jerks, long latencies of vertical antisaccades, a high error rate in the horizontal antisaccade task, and made more errors than controls when pro- and antisaccades were mixed. Based on oculomotor performance, a direct differentiation between EP and PD was possible only by the velocity of horizontal saccades. All remaining metrics were similar between both patient groups. EP patients present extensive oculomotor disturbances probably due to manganese-induced damage to the basal ganglia, reflecting their role in oculomotor system.

## Introduction

Ephedrone is a home-made psychostimulant drug used frequently in the former Soviet Union. This drug is prepared from over-the-counter common cold tablets containing ephedrine or pseudoephedrine, by oxidation with potassium permanganate in presence of acetic acid, without any further purification [1], containing a high residual manganese in the final synthetic mixture [2]. As a consequence, ephedrone addicts may show extremely high manganese (Mn) blood concentrations [3] and develop a chronic manganic encephalopathy similar to the one seen in manganese ore miners and in welders. This so called ephedrone-induced parkinsonism (EP) consists of a severe, rapidly progressive, irreversible and non-levodopa responsive parkinsonian and dystonic syndrome characterized by speech disorder [4], early gait impairment and postural instability [1], [3], [5]–[10]. Several studies have shown that in EP, prominent lesions occur in the GPi and substantia nigra pars reticulata (SNr), but recent evidence suggests more widespread neuropathology. Investigations in chronic Mn-intoxicated monkeys and welders with Mn intoxication have shown lesions affecting the substantia nigra pars compacta [11], brainstem, cerebellum [12], frontal white matter and cortical structures [8], [13].

Eye movements in EP have been reported to be slow and mildly restricted in the vertical and horizontal plane [1], [5], [9], [14], however they have never been objectively studied with videooculography. The role of the basal ganglia in the control of eye movements has been supported by extensive evidence [15]–[17]. In EP, Mn is the most likely etiological agent for both clinical symptoms and MR image changes, which can be observed as hyperintensive signal in T1-weighted MRI in the globus pallidus and in other basal ganglia (BG) structures such as the substantia nigra, caudate, and putamen [18]. With regard to the high representation of eye movement-related neurons in the BG [17], we hypothesized that BG damage due to Mn accumulation in EP can cause more serious dysfunction of eye movement control than in PD.

The aim of the present study was to analyse potential oculomotor abnormalities in EP patients by the use of video-oculography (VOG) and to compare these findings with VOG results in PD patients and healthy subjects.

## Methods

### Subjects

Patient characteristics are shown in Table 1.All participants signed the informed consent. The study was approved by the local ethics committees of the 1st Faculty of Medicine and General University Hospital, Prague, Czech Republic and of the S. Khechinashvili University Hospital, Tbilisi, Georgia and was in compliance with the Declaration of Helsinki.

**Table 1:** Clinical characteristics of EP and PD patients.

Pat	Gender/Age	DD	Treatment	NNIPPS T	NNIPPS OM	Pat	Gender/Age	DD	Treatment levodopa	Park2	UPDRS III	H&Y	NNIPPS OM
EP	M-F/years	Years	mg	/332	/21	PD	M-F/years	years	mg		/72	/5	/21
1	M44	6	-	23	0	1	M48	6	300	-	36	2	0
2	M48	4	-	52	6	2	M53	10	300	-	28	2	0
3	M40	6	-	62	6	3	M64	21	480	-	29	2	1
4	M28	4	-	37	3	4	M52	13	2535	normal gene	14	2	0
5	M44	7	-	59	8	5	M60	7	300	-	27	2	1
6	M41	6	-	29	2	6	M49	12	300	Polymorp.V380L	35	2	0
7	M39	10	-	72	3	7	M66	3	400	-	21	1	0
8	M42	4	-	38	2	8	F40	4	360	normal gene	16	1	1
9	M43	4	Levodopa 750	63	9	9	M44	7	870	normal gene	38	2	1
10	M35	6	-	33	1	10	F70	11	1050	-	25	1	1
11	M42	7	-	28	4	11	M54	12	900	-	8	2	0
12	M38	6	-	62	5	12	M58	7	400	-	20	1	0
13	M32	7	Trihexyphenidyl 19	90	2	13	F42	4	480	normal gene	47	3	0
14	M40	5	Levodopa 571	75	6	14	F48	10	450	normal gene	39	3	0
15	M42	5	Levodopa 71	34	5	15	M65	26	600	Polymorph. D394N	35	3	0
16	M32	4	-	36	1	16	F71	1	0	-	12	2	0
17	M46	4	-	47	6	17	M53	12	320	-	36	2	1
18	M44	2	-	43	8	18	M56	15	2620	-	26	2	0
19	M35	4	-	45	5	19	F63	11	320	-	17	2	1
20	M43	4	-	41	4	20	F42	6	100	normal gene	11	1	0
21	M31	12	-	83	4	21	F43	4	100	normal gene	24	3	0
22	M44	44	-	37	6								
23	F45	4	-	80	11								
24	M36	3	-	27	8								
25	M40	7	-	41	4								
26	M37	4	-	42	4								
27	M37	2	EDTA 20	21	5								
28	M40	6	-	88	6								
	27M-1F/40	6,68		49,57	4,79		13M-8F/54	9,62	627,86		25,9	1,95	0,33

Levodopa treatment indicates the dose in mg of levodopa or equivalent of dopamine agonist per day (0.7 mg pramipexole  = 100 mg levodopa; 5 mg ropinirole  = 100 mg levodopa). Patients treated with levodopa were examined in the “on” condition. The Park2 gene was evaluated for mutation if the age at disease onset was less than 40 years. Pat: patient number; EP: ephedrone parkinsonism; PD: Parkinson's disease; Age: age at examination in years; F: female; M: male; DD: disease duration; NNIPPS: neuroprotection and natural history in Parkinson plus syndromes; OM score: oculomotor score; MDS-UPDRS: movement disorder society-sponsored revision of the unified Parkinson's disease rating scale; H&Y: Hoehn and Yahr scale; EDTA: ethylenediaminetetraacetic acid.

EP patients: 28 patients (27 males, 1 female; mean age 39.9, SD 5.0, range 28.6–48.7 years) were examined at the department of neurology, S. Khechinashvili University Clinic, Tbilisi Georgia. The diagnosis of EP was based on a history of ephedrone use and subsequent development of a parkinsonian syndrome, with MRI showing pallidal hyperintensities on T1-weighted images in all patients. However, at the time of the present study, none of the patients were active consumers of ephedrone or other illicit drugs. The study was performed after the patients had stopped ephedrone consumption in average 3.9 years before the examination (range, 3 months to 12 years from stopping the drug use). A new 3T MRI was performed 2–3 weeks prior to the clinical examination (Magnetom Verio, SIEMENS) at the Research Institute of Clinical Medicine, Tbilisi, Georgia. Standard T1 (se), T2 (tse), FLAIR, T2*, and MPRAGE sequences were used for structural imaging. Only one patient (EP 27), who stopped ephedrone consumption 3 months before inclusion, showed typical bilateral diffuse hyperintensity on T1-weighted images in the globus pallidus (GP) and partially in the substantia nigra (SN). In all other cases, no pathological T1-hyperintensity was observed. Manganese concentration was measured in body and scalp hair at Uppsala University, Sweden (JB). Mean Mn concentration in our patients (0.50, SD 0.50 ppm) was well under the values obtained in the same laboratory for Estonian (0.82, SD 1.01 ppm Mn) and Swedish controls (0.83, SD 1.22 ppm Mn), confirming the absence of ongoing ephedrone use in EP patients. Patients were examined with the Natural History and Neuroprotection in Parkinson Plus Syndromes–Parkinson plus scale (NNIPPS) [19] to objectively assess parkinsonian-dystonic features and eye movement abnormalities. Neuropsychological testing consisted of the mini-mental state examination (MMSE) (mean 27.3/30), Beck Depression Inventory (BDI) (mean 19.1/64) and Frontal Assessment Battery (FAB) (mean 14.8/18).

PD control group: The group consisted of 21 patients (13 males, 8 females; mean age 54.8, SD 9.6, range 40–71 years) diagnosed according to the UK Parkinson's Disease Society Brain Bank criteria [20]. Patients younger than 40 years were genetically tested for the parkin (PARK2) mutation, and no carriers were found. All patients were examined at the Department of Neurology and Centre of Clinical Neuroscience, Charles University in Prague. The part III of the MDS-UPDRS [21] and Hoehn & Yahr [22] scales were used for clinical evaluation. Additionally eye movements were examined using the oculomotor part of the NNIPPS-Parkinson plus scale. Neuropsychological testing included the MMSE (mean 27.6/30), BDI (mean 10.3/64) and FAB (mean 16/18).

Healthy control group: The control group was included to establish a normal baseline and consisted of 27 participants (25 males, 2 females; mean age 36.2, SD 6.0, range 26–45 years), MMSE (mean 28.9/30), BDI (mean 4.9/64), FAB (mean 17.7/18). A questionnaire was used to determine that all controls were free of any neurological or psychiatric illness, and all controls denied the intake of any medication acting on the central nervous system.

### Oculomotor examination

Eye movements were examined in all subjects by the same investigator (CB) using a binocular video-based eye tracker (mobile eBT Eye brain, Ivry-sur-Seine, France, www.eye-brain.com, 300 Hz sampling rate and 0.5° spatial resolution).Saccades were automatically detected according to a velocity threshold (Eye brain software) but were individually inspected and manually corrected by the experimenter if necessary. The left eye trace was analyzed by default, however the right eye was used if the left eye signal was contaminated by artifacts. Saccades perturbed by blinks or other artifacts were discarded (less than 10% of the trials in all subjects). Saccades with a latency below 80 ms were considered anticipatory saccades and rejected, and SRT between 81 and 130 ms were considered “express saccades” [23].

Three different tasks were performed in the same order in one session of 30 minutes duration: i) Simple prosaccades horizontal and vertical; ii) Simple antisaccades horizontal and vertical; iii) Mixed horizontal pro- and antisaccades. Subjects were seated in a calm, dark room with their chin supported by a chin strap and their forehead in contact with a frontal support. They faced a flat, 26 in. LCD screen (ProLite, Iiyama model PL 2600, size 550 mmx344 mm) located 60 cm in front of them at eye level.

- Simple horizontal and vertical prosaccades: This task started with the onset of a green central fixation point(size: 15×15 pixels; luminance: 120 cd/m2) that was presented for a pseudorandom duration of 2800, 3200, 3500, 3800, 4000 or4100 ms. The fixation point was then turned off and 200 ms later, a red peripheral target (15×15 square, luminance 120 cd/m2) appeared during 1000 ms at a 13°right or left location, or at a 13°up or down location. Twenty-eight saccades were recorded. Latency, velocity [average (Vavg) and maximal (Vmax)] and gain were analyzed for each saccade. Then an average of all saccades for each metric was performed in each patient. Latency was defined as the reaction time from the target onset to begin of the saccade. Gain was defined as the ratio between saccade amplitude and target location. The number and amplitude of square wave jerks (SWJs) were measured during the period when the fixation point was on, lasting for 56 seconds. Square-wave jerks are small, inappropriate saccades that intrude on steady fixation by taking the eye away from the target and then returning it to the fixation position [24]. Only horizontal SWJs between 1–10° were considered for analysis, because SWJ over 10° are considered macro SWJ [25].
- Simple horizontal and vertical antisaccades: The task design was the same as in the prosaccade task, with the exception that the color of the central fixation point was red. Subjects were instructed to look as fast as possible in the direction opposite to the peripheral target. A total number of 32 saccades were recorded. Latency, error rate and rate of corrected errors were extracted. Saccades perturbed by blinks or other artefacts were discarded (less than10% of the trials in all subjects). In the pro-and antisaccade tasks, we defined the latency as the interval between target onset and saccade onset. Latency below 80 ms were considered anticipatory saccades and rejected [23]. Mean latency was determined only for correct antisaccades. Directional errors were defined as saccades initially directed towards the target. The rate of corrected errors (%) was extracted for the horizontal antisaccade task.
- Mixed task of pro- and antisaccades: This paradigm, performed according to Rivaud-Pechoux [26], was used to evaluate the ability to perform a task in which two task sets, rather than one, must be handled simultaneously, thereby demanding an increased cognitive load, increased demands on working memory, vigilance, sustained attention, motivation and response selection [26]. The central fixation point initially consisted of two vertically aligned and contiguous red and green points, with the same size and luminance as in the two previous task. After 3500–4200 ms, one of the two points (red or green) was turned off. The remaining point stayed on for 500 ms, and subjects were instructed that the color of the fixation point was to be used for selecting the appropriate response to the lateral target: a green point required a prosaccade and a red point anantisaccade. A 200 ms gap between the fixation point and the lateral target was used as in the previous tasks. We confirmed verbally that the instructions had been correctly understood. Seven prosaccades and six antisaccades were presented with an angle of 24°. In each subject, we calculated mean pro- and antisaccade latencies and error rates in the antisaccade task. Then we selectively analyzed saccades repeated in the same direction. Repeated trials were analyzed to provide a mixing cost for latencies and error rates, defined as performance. The performance in repeated trials was subtracted from the performance in the simple tasks of horizontal pro and antisaccades. We employed the restrictive method of analysis of Rivaud-Pechoux [26], taking into account only N-1 trials executed correctly with the same instructions. We first analyzed results separately to the right and left direction, and then as there were no differences between both sides, we elected to pool right and left pro/antisaccades.

### Statistical analysis

Matlab© (Mathworks, Massachusetts, USA) was used for statistical analyses. As the Kolmogorov-Smirnov test for independent samples did not detect abnormal distribution of oculomotor variables, analysis of variance (ANOVA) was used to assess differences between the EP and healthy control group. Since the PD patients were generally older when compared to EP subjects, analysis of covariance (ANCOVA) was used to calculate differences between EP and PD groups with age as a covariate. The Pearson correlation analysis was used to examine the relationships between eye metrics and clinical and neuropsychological data. Post-hoc Bonferroni adjustment was applied to correct for the number of all tests performed according to the each paradigm. The level of significance after Bonferroni adjustment was set to *p*<0.05.

## Results

**Figure 1:**
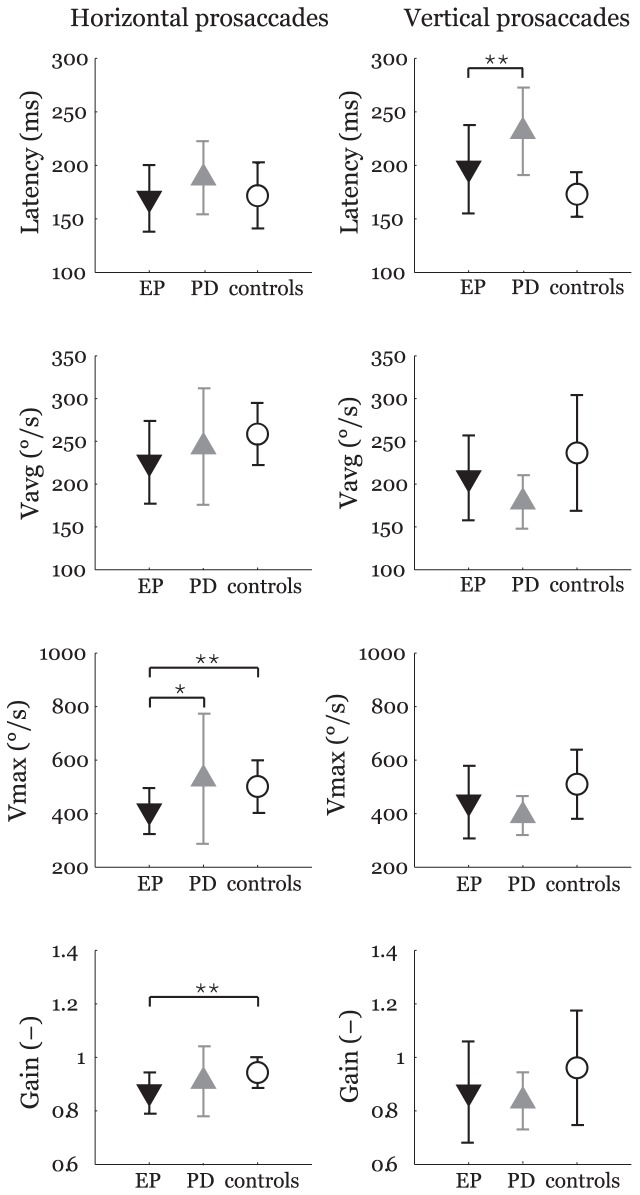
Latencies, average velocities (Vavg), maximal velocities (Vmax), and gains for horizontal (left) and vertical (right) prosaccades. Comparison of EP patients with PD and healthy control groups after Bonferroni adjustment: *p<0.05; **p<0.01; ***p<0.001. The symbols represent mean values and error bars standard deviations. EP  =  ephedrone parkinsonism; PD  =  Parkinson's disease.

The clinical data of EP as well as PD patients can be seen in Table 1.

i. Simple prosaccades (Figure 1.): In horizontal prosaccades, EP patients showed significantly decreased Vmax (*F*_1,54_ = 13.3, *p* = 0.005, *η* ^2^ = 0.20), significantly lower gain (*F*_1,54_ = 16.0, *p* = 0.002, *η* ^2^ = 0.24), a trend toward decrease Vavg (*F*_1,54_ = 8.0, corrected *p* = 0.06, uncorrected *p* = 0.007, *η* ^2^ = 0.13), and normal latency (*F*_1,54_ = 0.09, *p* = 1.00, *η* ^2^ = 0) as compared to controls. In addition, EP patients showed decreased Vmax (*F*_1,44_ = 10.2, *p* = 0.02, *η* ^2^ = 0.23) in comparison to PD subjects. There were no differences between PD and EP patients regarding latency (*F*_1,44_ = 4.1, *p* = 0.43, *η* ^2^ = 0.09), Vavg (*F*_1,44_ = 4.5, *p* = 0.36, *η* ^2^ = 0.10), and gain (*F*_1,44_ = 0.2, *p* = 1.00, *η* ^2^ = 0.01).

In vertical prosaccades, when compared to controls, EP patients showed a trend toward longer latency (*F*_1,54_ = 7.8, corrected *p* = 0.07, uncorrected *p* = 0.007, *η* ^2^ = 0.13) whereas other eye movement metrics including Vavg (*F*_1,54_ = 3.3, *p* = 0.66, *η* ^2^ = 0.06), Vmax (*F*_1,54_ = 3.5, *p* = 0.60, *η* ^2^ = 0.06), and gain (*F*_1,54_ = 2.6, *p* = 1.00, *η* ^2^ = 0.05) remained normal. In comparison to PD subjects, EP patients manifested significantly shorter latency (*F*_1,44_ = 13.8, *p* = 0.005, *η* ^2^ = 0.31) whereas no differences in Vavg (*F*_1,44_ = 0.1, *p* = 1.00, *η* ^2^ = 0), Vmax (*F*_1,44_ = 0.3, *p* = 1.00, *η* ^2^ = 0.01), and gain (*F*_1,44_ = 1.4, *p* = 1.00, *η* ^2^ = 0.03) were observed.

**Figure 2:**
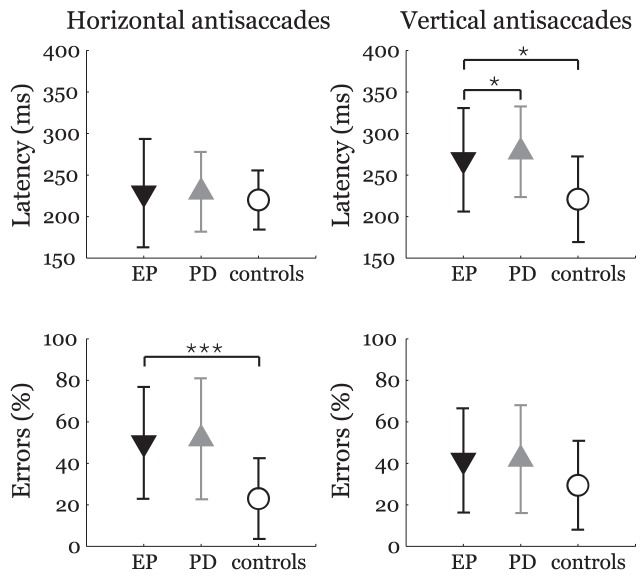
Latencies and error rates for horizontal (left) and vertical (right) antisaccades. Comparison of EP patients with PD and healthy control groups after Bonferroni adjustment: *p<0.05; **p<0.01; ***p<0.001. The symbols represent mean values and error bars standard deviations. EP  =  ephedrone parkinsonism; PD  =  Parkinson's disease.

Considering square wave jerks, EP patients produced more SWJs (EP mean number 6.79, SD 6.72, controls mean number 2.26, SD 3.98; *F*_1,54_ = 9.2, *p* = 0.03, *η* ^2^ = 0.15) than controls but no difference in SWJ between EP and PD groups were observed (PD mean number 6.38, SD7.34; *F*_1,44_ = 0.3, *p* = 1.00, *η* ^2^ = 0.01).

ii. Simple antisaccades (Figure 2): In horizontal direction, EP patients produced more errors than controls (*F*_1,54_ = 17.8, *p*<0.001, *η* ^2^ = 0.25) while there was no significant difference for latency (*F*_1,54_ = 0.3, *p* = 1.00, *η* ^2^ = 0.01). No significant differences were noted between PD and EP groups for both latencies (*F*_1,44_ = 0.6, *p* = 1.00, *η* ^2^ = 0.01) and errors (*F*_1,44_ = 0.9, *p* = 1.00, *η* ^2^ = 0.02).

**Figure 3:**
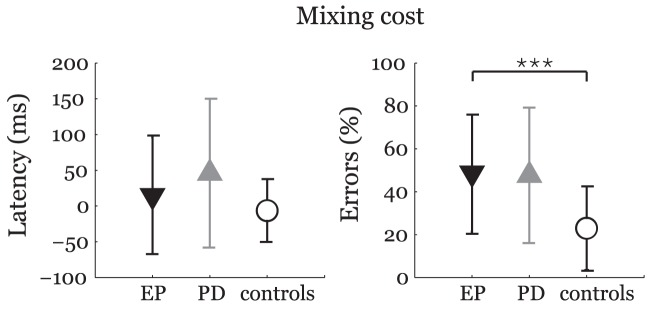
Latency and error rate for mixing cost. Comparison of EP patients with PD and healthy control groups after Bonferroni adjustment: *p<0.05; **p<0.01; ***p<0.001. The symbols represent mean values and error bars standard deviations. EP  =  ephedrone parkinsonism; PD  =  Parkinson's disease.

In vertical direction, latency was found to be longer for EP group when compared to controls (*F*_1,54_ = 16.5, *p* = 0.01, *η* ^2^ = 0.15) whereas error rate remained unaffected (*F*_1,54_ = 3.6, *p* = 0.25, *η* ^2^ = 0.06). Interestingly, EP patients manifested significantly shorter latencies when compared to PD subjects (*F*_1,44_ = 10.1, *p* = 0.01, *η* ^2^ = 0.22). There was no difference between EP and PD group for error rate (*F*_1,44_ = 0.1, *p* = 1.00, *η* ^2^ = 0). EP patients showed a rate of movement correction after an incorrect antisaccade of 93%.

iii. Mixed task of pro- and antisaccades (Figure 3 details the results of mixing cost for the latency and error rate of antisaccades): There was increased error rate in EP group when compared to controls (*F*_1,54_ = 15.6, *p*<0.001, *η* ^2^ = 0.23), whereas no differences were found for latency (*F*_1,54_ = 1.3, *p* = 0.50, *η* ^2^ = 0.03). No differences between EP and PD groups were seen for both latency (*F*_1,44_ = 0.2, *p* = 1.00, *η* ^2^ = 0.01) and error rate (*F*_1,44_ = 0, *p* = 1.00, *η* ^2^ = 0).

No correlations were found between the neuropsychological assessment scores and eye movement metrics in EP patients.

## Discussion

Ephedrone patients, in comparison to healthy controls, had slow and hypometric horizontal saccades, long latencies of vertical antisaccades, a high error rate in the horizontal antisaccade task, more errors than controls when pro- and antisaccades were mixed, and an increased occurrence of square wave jerks. The only direct significant difference between EP and PD concerned a slower peak velocity of horizontal saccades in EP. Yet, the latency for both vertical prosaccades and antisaccades was prolonged in EP when compared to healthy controls. In particular, an isolated prolongation of latency of vertical, but not horizontal saccades, has to the best of our knowledge, not been observed previously. This difference suggests that the saccade reaction time may be driven independently in the horizontal and vertical plane, and highlights again the importance of studying EM in both directions [27]. In general terms the latency of saccades has been related to bilateral [28] activation of the posterior parietal and frontal cortices [29]. Nevertheless a study by Kaneko implicates also subcortical structures in the control of this metric, showing in the pharmacologically-inactivated nucleus reticularis tegmenti pontis of the monkey brain, unusually long latency of vertical saccades [30].

Horizontal prosaccades were slower and hypometric when comparing EP patients with controls, while the latency was preserved [29], [31]. Slow and hypometric prosaccades are also hallmarks of patients with hereditary ataxias, vascular lesions at the pons and cerebellum, Gaucher's disease Type 3 and Tay-Sachs disease [29], [32]. However, in those disorders, saccades seem to be considerably slower, clinically and in recordings. The velocity of horizontal saccades has been related to the prepontine reticular formation [31], while the accuracy, a less specific eye movement measure, may be distorted in disorders of the cerebellum, brainstem and peripheral oculomotor pathways [29].

EP patients presented an increased number of SWJ during saccade tasks. The pathophysiology of SWJs is unknown, but they have been related to disruption of cerebral, cerebellar, basal ganglia function [33], [34] and specifically in lesions of the GP [16], [33], [35]. High number of SWJ has been previously reported in PD [36], after unilateral pallidotomy [16], [37], or stimulation of the nucleus subthalamicus [38], and they have also been found in progressive supranuclear palsy (PSP) [39]. Similar to PSP, EP patients show gait and speech disturbances, and a non levodopa responsive parkinsonian syndrome. However, in PSP the predominant eye movement defects concern slow and hypometric vertical saccades [40], while those metrics were mostly preserved in our EP group.

Both in our EP and PD patients, the antisaccade error rate was increased for horizontal, but not for vertical antisaccades. To our knowledge, such dissociation between high error rates in the horizontal and not in the vertical plane has not been described before. These changes are not related to age since we demonstrated in a previous study that both metrics increase with age but not in a dissociated manner [27]. In humans and non-human primates, the dorsolateral prefrontal cortex (DLPFC) has been related to inhibition of reflexive saccades [41]. Impaired inhibition of reflexive horizontal saccades has been described in PSP patients associated with the involvement of the DLPFC in the degenerative process [42]. Recent non-human primate studies suggest that the GP might regulate eye movements through the nigro-collicular descending circuitry, via the basal ganglia thalamocortical pathways, playing an important role in suppressing inadequate antisaccades [43]. Consequently, a specific involvement of the GP might underlie the increased antisaccade error rate in EP patients [44] but it does not explain the dissociation between horizontal and vertical antisaccade direction.

In addition, our EP patients exhibit an increased error rate when pro and antisaccades were mixed. Mixing costs for pro- and antisaccade error rates were low in our control group, in agreement with previous studies [45], [46] whereas it was increased in PD as previously described [47], without significant difference to EP patients. The increased mixing cost has been associated to recruitment of additional cerebral structures as the supplementary eye field [48], leading to the hypothesis that its activation may partially reflect task shifting [49], [50].

As already mentioned, the only significant difference in oculomotor performance between EP and PD concerned peak horizontal saccade velocity. It may reflect a distinct impairment of specific neural networks underlying the pathology of EP.

A homozygous mutation of the Mn transporter SLC30A10 causing severe hypermanganesemia, dystonia, parkinsonism, polycythemia, and chronic hepatic disease has recently been described [51]. SLC30A10 is highly expressed in the GP, subthalamic nucleus, putamen, deep cerebellar nuclei, and other diencephalic and cortical areas [51]. At the annual meeting of the American Academy of Neurology in 2013, Pretegiani and Rufa [52] presented two cases of SLC3A10 mutations with eye movement abnormalities similar to those found in our EP patients, including slow and hypometric horizontal saccades, but also a high error rate in the antisaccade task. This suggests that manganese toxicity may be the determining factor in the pathogenesis of eye movement abnormalities in EP.

There were no correlations found in our data set between the VOG metrics and severity of eye movement abnormalities as rated by the oculomotor part of the NNIPPS. We chose the NNIPPS as it is the only available clinical scale that includes eye movement evaluation in patients with atypical parkinsonian syndromes. However, NNIPPS allows to semiquantitatively rate only amplitude and speed of voluntary horizontal and vertical saccades. Therefore it may not be sufficiently sensitive to reliably capture distinct but discrete oculomotor abnormalities observed using VOG in our EP group. In particular, latencies and error rates of antisaccades were clearly abnormal in EP but their evaluation is not contained in the NNIPPS. Anyhow, this highlights the importance of incorporating VOG examination, as a sensitive non-invasive tool to reveal slight oculomotor changes. Furthermore, although eye movement performance has been shown to be correlated with UPDRS subscores [53], cognitive function in PD [54], [55] and/or verbal fluency [56], we did not reveal any correlation between the severity of neuropsychological impairment assessed with MMSE, BDI and FAB and EM metrics in our EP group. One possible explanation is that our EP patients manifested only very mild cognitive impairment and therefore a more specific neuropsychological assessment would be needed to reveal possible relationships between cognitive and eye movement functions.

In summary, the present study shows that eye movement abnormalities due to ephedrone abuse share similar features but also exhibit certain differences from PD. Similarly to PD patients, subjects with ephedrone-induced parkinsonism demonstrate decreased gain for horizontal prosaccades, increased occurrence of square wave jerks, long latencies of vertical antisaccades as well as a high error rate in the horizontal antisaccade task and when mixing pro- and antisaccades. On the other hand, aspects such as decreased peak velocity of horizontal saccades and affection of latencies only in vertical direction can correspond to pathogenic mechanisms of ephedrone-induced parkinsonism reflecting a specific involvement of globus pallidus and other brain structures due to manganese intoxication.
